# Coreference based event-argument relation extraction on biomedical text

**DOI:** 10.1186/2041-1480-2-S5-S6

**Published:** 2011-10-06

**Authors:** Katsumasa Yoshikawa, Sebastian Riedel, Tsutomu Hirao, Masayuki Asahara, Yuji Matsumoto

**Affiliations:** 1 Graduate School of Information Science, Nara Institute of Science and Technology, 8916-5 Takayama, Ikoma, Nara, Japan; 2 University of Massachusetts, Amherst, Amherst, MA 01002, U.S; 3 NTT Communication Science Laboratories, 2-4, Hikaridai, Seika-cho, Keihanna Science City, Kyoto, Japan

## Abstract

This paper presents a new approach to exploit coreference information for extracting event-argument (E-A) relations from biomedical documents. This approach has two advantages: (1) it can extract a large number of valuable E-A relations based on the concept of *salience in discourse*; (2) it enables us to identify E-A relations over sentence boundaries (cross-links) using *transitivity* of coreference relations. We propose two coreference-based models: a pipeline based on Support Vector Machine (SVM) classifiers, and a joint Markov Logic Network (MLN). We show the effectiveness of these models on a biomedical event corpus. Both models outperform the systems that do not use coreference information. When the two proposed models are compared to each other, joint MLN outperforms pipeline SVM with gold coreference information.

## Introduction

The increasing amount of biomedical texts resulting from high throughput experiments demands the automatic extraction of useful information by Natural Language Processing techniques. One of the more recent information extraction tasks is biomedical event extraction. With the introduction of the GENIA Event Corpus [1] and the BioNLP’09 shared task data [2], a set of documents annotated with events and their arguments, various approaches for event extraction have been proposed so far [3-5].

Previous work has considered the problem on a per-sentence basis and neglected possibly useful information from other sentences in the same document. In particular, no one has yet considered using coreference information to improve event extraction. Here we propose a new approach to extract event-argument (E-A) relations that does make use of coreference information.

Our approach includes two main ideas:

1. extracting coreferent arguments based on *salience in discourse*

2. predicting arguments over sentence boundaries with the help of a *transitivity* relation.

**Figure 1:**
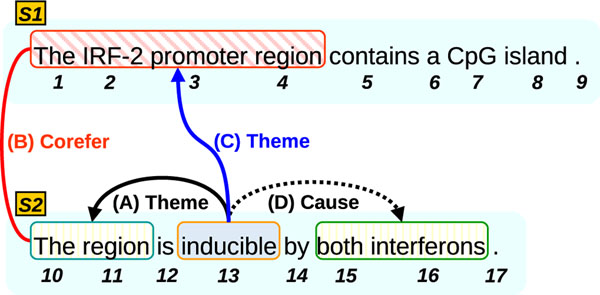
**Cross-sentence event-argument relation.** An example of event-argument relation crossing sentence boundaries. In this figure, an event, “inducible” has “The region” as an Theme. But “The region” is coreferent to “The IRF-2 promoter region” in the forward sentence. So, “The IRF-2 promoter region” is also a Theme of “inducible”.

First, noun phrases (NPs) that corefer with other NPs have an implicit significance in discourse structures based on Centering Theory [6]. Significant entities are highly likely to be mentioned multiple times. We call this kind of significance ”*salience in discourse*.” *Salience in discourse* is a useful criterion for measuring the importance of entity mentions, and this criterion gives our E-A relation extractors a higher chance to extract arguments which are coreferent with other mentions. When considering discourse structure, arguments which are coreferent to something (e.g. “The region” in Figure 1) also have higher *salience in discourse*. They are hence more likely to be arguments of other events mentioned in the document. Using this information helps us to identify the correct arguments for candidate events and increases the likelihood of extracting arguments with antecedents corresponding to the Arrow (A) in Figure 1. Note that identifying coreferent arguments is not just important to improve the F1 score of event-argument relation extraction: assuming that *salience in discourse* indicates the novel information the author wants to convey, these are the arguments we should extract at any cost.

Secondly, *transitivity* is a property of event-argument relations such that the relation between an event and its argument is transitive across coreference relations. It enables us to extract cross-sentence mentions as arguments of events. Previous work on this task has primarily focused on identifying event-arguments within a sentence. However cross-sentence event-argument relations are common, for example see Figure 1. It illustrates an example of E-A relation extraction including cross-sentence E-A. In the sentence *S2*, we have “inducible” as an event to be identified. When identifying intra-sentence arguments in *S2*, we obtain “The region” as Theme and “both interferons” as Cause.

However, in this example, “The region” is not optimal as a Theme because “The region” is coreferent to “The IRF-2 promoter region” in *S1*. Thus, the true Theme of “inducible” is “The IRF-2 promoter region” as this phrase is more informative as an argument. In this case, “The region” is just an anaphor of the true argument. The idea of *transitivity* entails that if “The region” is a Theme of “inducible” and “The region” is coreferent to “The IRF-2...”, then “The IRF-2...” is also a Theme of “inducible”. It allows us to extract cross-sentence E-A relations such as the Arrow (C) in Figure 1.

We propose two models which implement these ideas to extract event-argument (E-A) relations involving coreference information. One is based on local classification with SVM, and another is based on a joint Markov Logic Network (MLN). To remain efficient, and akin to existing approaches, both look for events on a per-sentence basis. However, in contrast to previous work, our models consider as candidate arguments not only the tokens of the current sentence, but also all tokens in the previous sentences that are identified as antecedents of some tokens in the current sentence. We show the effectiveness of our models on a biomedical corpus. They enable us to extract cross-sentence E-A relations: We achieve an F1 score of 69.7% in our MLN model, and 54.1 % in the SVM pipeline. Moreover, with the idea of *salience in discourse* our coreference-based approach helps us to improve intra-sentence E-A extraction, in particular when arguments have antecedents. In this case adding gold coreference information to MLNs improves F-score by 16.9%. In place of gold coreference information, we also experiment with predicted coreferences from a simple coreference resolver. Although the quality of predicted coreference information is relatively poor, we show that using this information is still better than not using it at all.

## Background

### Biomedical event extraction

**Figure 2:**
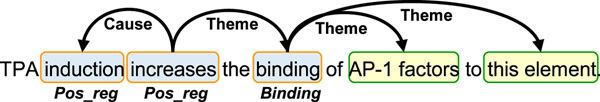
**Biomedical event extraction.** A simple example of biomedical event extraction. Event: induction, increases, binding. Argument: AP-1 factors, this element, induction, binding Role: increases - induction (Cause), increases - binding (Theme), binding - AP-1 factors (Theme), binding - this element (Theme)

Event extraction on biomedical text involves three sub-tasks; identification of event trigger words; classification of event types; extraction of the arguments of the identified events (E-A). Figure 2 shows an example of event extraction. In this example, we have three event triggers: “induction”, “increases”, and “binding”. The corresponding event types are *Positive_regulation* (*Pos_reg*) for “induction” and “increases”, and *Binding* for “binding”. In Figure 2, “increases” has two arguments; “induction” and “binding”. The roles we have to identify fall into two classes: “Theme” and “Cause”. In the case of our example the roles of the two arguments of “increases” are Cause and Theme, respectively. Note that in biomedical corpora a large number of nominal events can be found. For example, in Figure 2 the arguments of “increases” are both nominal events. Such events which are arguments of other events are often hard to identify.

### Biomedical corpora for event extraction

There are two major corpora for biomedical event extraction: The GENIA Event Corpus (GEC) [1], and the data of the BioNLP’09 shared task (http://www-tsujii.is.s.u-tokyo.ac.jp/GENIA/SharedTask/). The latter is in fact derived from the GEC. There are some important differences between them.

**event type** GEC has fine-grained event type annotations (35 classes), while BioNLP’09 data focuses on only 9 event subclasses.

**non-event argument** BioNLP’09 data does not differentiate between protein, gene and RNA, while the GEC corpus does.

**coreference annotation** Both GEC and BioNLP’09 corpora provide coreference annotations related to event extraction. However, in the case of the BioNLP’09 data coreference information primarily concerns protein names and abbreviations that follow in parenthesis. The GEC, on the other hand, provides proper cross-sentence coreference. Moreover, the sheer number of coreference annotations is much larger. Björne et al. [3] also mentioned that coreference relations could be helpful for cross-sentence E-A extraction but the coreference annotation necessary to train a coreference resolver is not present in BioNLP’09 data.

For our work we choose the GEC, primarily because of the amount and quality of coreference information it provides. This allows us to train a coreference resolver, as well as testing our hypothesis when gold coreference annotations are available. The second reason to prefer GEC over the BioNLP’09 corpus is its fine-grained annotation. We believe that this setting is more realistic.

### Issues of previous work

Various approaches have been proposed for event-argument relation extraction on biomedical text. However, even the current state-of-the-art does not exploit coreference relations and focuses exclusively on intra-sentence E-A extraction.

BioNLP’09 has three tasks 1, 2, and 3. Task 1 is core event extraction and mandatory. Our work also focuses on Task 1. For example, Björne et al. achieved the best results for Task 1 in the BioNLP’09 competition [3]. However, they neglected all cross-sentence E-A. They also reported that they did try to detect cross-sentence arguments directly without the use of coreference. This approach did not lead to a reasonable performance increase.

In BioNLP’09, Riedel et al. proposed a joint Markov Logic Network to tackle the task, and achieved the best results for Task 2 [7]. Their system makes use of global features and constraints, and performs event trigger and argument detection jointly. Poon and Vanderwende [5] also applied Markov Logic and achieved competitive performance to the state-of-the-art result of Björne [3]. However, in both cases no cross-sentence information is exploited. To summarize, so far there has been no research within biomedical event extraction which exploits coreference relations and tackles cross-sentence E-A relation extraction. By contrast, for predicate-argument relation extraction in a Japanese newswire text corpus (http://cl.naist.jp/nldata/corpus/), Taira et al. do consider cross-sentence E-A extraction [8]. However, they directly extract cross-sentence links without considering coreference relations. Moreover, their approach is based on a pipeline of SVM classifiers, and their performance on cross-sentence E-A extraction was generally low (Low 20s% F1).

### The direction of our work

We present a new approach that exploits coreference information for E-A relation extraction. Moreover, in contrast to previous work on the BioNLP’09 shared task we apply our models in a more realistic setting. Instead of relying on gold protein annotations, we use a Named Entity tagger; and instead of focusing on the coarse-grained annotation of the BioNLP task, we work with the GEC corpus and its fine-grained ontology.

From now on, for brevity, we refer to cross-sentence event-argument relations as “*cross-links*” and intra-sentence event-argument relations as “*intra-links*”.

We propose two coreference-based models. One is an SVM based model that extracts intra-links first and then cross-links as a post-processing step. The other is a joint model defined with Markov Logic that jointly extracts intra-links and cross-links and allows us to model salience of discourse in a principled manner.

## Methods

We have two ideas for incorporating coreference information into E-A relation extraction:

• Extracting valuable E-A relations based on “*salience in discourse*”

• Predicting cross-links by using “*transitivity*” including coreference relations

*Salience in discourse* is the idea of considering how important the occurring mentions are. We exploit it as a likelihood of arguments of events. *Transitivity* is a property of event-argument relations such that the relation between an event and its argument is transitive across coreference relations. It enables us to identify the E-A relations over sentence boundaries. According to these ideas, we propose two approaches. One is a pipeline model based on SVM classifiers, and the other is a joint model based Markov Logic.

### SVM pipeline model

In our pipeline model we apply the SVM model proposed by [3]. Their original model first extracts events and event types with a multi-class SVM (1st phase). Then it identifies the relations between all event-protein and event-event pairs by another multi-class SVM (2nd phase). Note that in our setting, the 1st phase classifies event types into 36 classes (35 types + “Not-Event”). Moreover, while protein annotations were given in the BioNLP’09 shared task, for the GEC we extract them using an NE tagger. The features we used for the 1st and 2nd phases are summarized in the first and the second columns of Table 1, respectively.

**Table 1:** Used local features for SVM pipeline and MLN joint

Description	SVM 1st phase *event* &*eventType*	SVM 2nd phase *role* (E-A)	MLN predicate
Word Form	X	X	*word*(*i*,*w*)
Part-of-Speech	X	X	*pos*(*i*, *p*)
Word Stem	X	X	*stem*(*i*, *s*)
Named Entity Tag	X	X	*ne*(*i*,*n*)
Chunk Tag	X	X	*chunk*(*i*, *c*)
In Event Dictionary	X	X	*dict*(*i*, *d*)
Has Capital Letter	X	X	*capital*(*i*)
Has Numeric Characters	X	X	*numeric*(*i*)
Has Punctuation Characters	X	X	*pun*()
Character Bigram	X		*bigram*(*i*, *bi*)
Character Trigram	X		*trigram*(*i*, *tri*)

Dependency label	X	X	*dep*(*i*, *j*, *d*)
Labeled dependency path between tokens		X	*path*(*i*, *j*, *pt*)
Unlabeled dependency path between tokens		X	*pabhNL*(*i*, *j*, *pt*)
Least common ancester of dependency path		X	*lca*(*i*, *j*, *L*)

After identifying intra-links, the pipeline model deterministically attaches, for each intra-sentence argument of an event, all antecedents inside/outside the current sentence. We implement *transitivity* as a post-processing step. However, it is difficult for the SVM pipeline to implement the idea of *salience in discourse*. We believe that a Markov Logic model is preferable in this case.

### MLN joint model

Markov Logic [9] is an expressive template language that uses weighted first-order logic formulae to instantiate Markov Networks of repetitive structure. In Markov Logic users design predicates and formulae to model their problem. Then they use software packages such as *Alchemy* (http://alchemy.cs.washington.edu/) and *Markov thebeast* (http://code.google.com/p/thebeast/) in order to perform inference and learning.

It is difficult to construct Markov Logic Networks for joint E-A relation extraction and coreference resolution across a complete document. Hence we follow two strategies: (1) restriction of argument candidates based on coreference relations; (2) construction of a joint model which collectively identifies intra-links and cross-links. Restricting argument candidates helps us to construct a very compact yet still effective model. A joint model enables us to simultaneously extract intra-links and cross-links and contributes to the performance improvement. In addition, we will see that this setup still allows us to implement the idea of *salience in discourse* with global formulae in Markov Logic.

#### Predicate definition

Our joint model is based on the model proposed by [7]. We first define the predicates of the proposed Markov Logic Network (MLN). There are three “*hidden*” predicates corresponding to what the target information we want to extract(Table 2).

**Table 2:** The three hidden predicates

event(*i*)	token *i* is an event
eventType(*i*, *t*)	token *i* is an event with type *t*
role(*i*, *j*, *r*)	token *i* has an argument *j* with role *r*

In this work, *role* is the primary hidden predicate since it represents event-argument relations. Next we define *observed* predicates representing information that is available at both train and test time. We define corefer(*i*, *j*), which indicates that token *i* is coreferent to token *j* (they are in the same entity cluster). corefer(*i*, *j*) obviously plays an important role in our coreference-based joint model. We list the remaining observed predicates in the last column of Table 1.

Our MLN is composed of several weighted formulae that we divide into two classes. The first class contains local formulae for *event*, *eventType*, and *role*. We say that a formula is local if it considers only one single hidden ground atoms. The formulae in the second class are global: they involve two or more atoms of hidden predicates. In our case they consider *event*, *eventType*, and *role* atoms simultaneously.

#### Basic local formulae

Our local features are based on features employed in previous work [3,7] and listed in Table 1. We exploit two types of formula representation: “simple token property” and “link tokens property” defined by [7].

The first type of local formulae describes properties of only one token and such properties are represented by the predicates in the first section of Table 1. The second type of local formulae represents properties of token pairs and linked tokens property predicates (*dep*, *path*, *pathNL*, and *lca*) in the second section of Table 1.

#### Basic global formulae

Our global formulae are designed to enforce consistency between the three *hidden* predicates and are shown in Table 3. Riedel et al. [7] presented more global formulae for their model. However, some of these do not work well for our task setting on the GENIA Event Corpus. We obtain the best results by only using global formulae for ensuring consistency of the hidden predicates.

**Table 3:** Basic global formulae

Formula	Description
*event*(*i*) ⇒ ∃*t.eventType*(*i*, *t*)	If there is an event there should be an event type
*eventType*(*i*, *t*) ⇒ *event*(*i*)	If there is an event type there should be an event
*role*(*i*, *j*, *r*) ⇒ *event*(*i*)	If *j* plays the role *r* for *i* then *i* has to be an event
*event*(*i*) ⇒ ∃*j.role*(*i*, *j*, *Theme*)	Every event relates to need at least one argument.

### Using coreference information

We explain our coreference-based approaches using the example in Figure 1. First, the two intra-links in *S2* are represented by role(13, 11, Theme) – Arrow (A) and role(13, 15, Cause) – Arrow (D). Note, in these terms, phrasal arguments are driven by *anchor* tokens which are the ROOT tokes on dependency subtrees of the phrases. The coreference relation is represented by corefer(11, 4) – Bold Line (B). Finally, the cross-link is represented by role(13, 4, Theme) – Arrow (C).

With the example in Figure 1, we explain the two main concepts : *Salience in Discourse* (*SiD*) and *Transitivity* (*T*). We also present an additional idea, *Feature Copy* (*FC*).

#### Salience in discourse

The entities mentioned over and over again are important in discourse and accordingly highly likely to be arguments of some events. In order to implement this idea of *salience in discourse*, we add the Formula (*SiD*), shown in the first row of Table 4. Formula (*SiD*) requires that if a token *j* is coreferent to another token *k*, there is at least one event related to token *j.* Our model with Formula (*SiD*) prefers coreferent arguments and aggressively connects them with events. Note that our coreference resolver always extracts coreference relations which are related to events, since coreference annotations in GEC are always related to events.

**Table 4:** Coreference formulae

Symbol	Name	Formula	Description
(*SiD*)	*Salience in Discourse*	corefer(*j*, *k*) ⇒ ∃*i.rol*e(*i*, *j*, *r*) ∧ event(*i*)	If a token *j* is coreferent to another token *k*, there is at least one event related to token *j*
(*T*)	*Transitivity*	role(*i*, *j*, *r*) ∧ corefer(*j*, *k*) ⇒ role(*i*, *k*, *r*)	If *j* plays the role *r* for *i* and *j* is coreferent to *k* then *k* also plays the role *r* for *i*
(*FC*)	*Feature Copy*	corefer(*j*, *k*) ∧ *F*(*k*, +*f*) ⇒ role(*i*, *j*, *r*)	If *j* is coreferent to *k* and *k* has feature *f* then *j* plays the role *r* for *i*

#### Transitivity

Another main concept is *“transitivity”*, which is important for intra/cross-link extraction. As mentioned earlier, the SVM pipeline enforces *transitivity* as a post-processing step. For the MLN joint model, let us consider the example of Figure 1 again.

role(13,11, Theme) ∧ corefer(11, 4) ⇒ role(13, 4, Theme)

This formula denotes that, if an event “inducible” has “The region” as a Theme and “The region” is coreferent to “The IRF-2 promoter region”, then “The IRF-2 promoter region” is also a Theme of “inducible”. The three atoms, role(13,11, Theme), corefer(11, 4), and role(13,4, Theme) in this formula correspond respectively to the three Arrows (A), (B), and (C) in Figure 1. This formula is generalized as Formula (*T*) shown in the second row of Table 4. The merit of using Formula (*T*) is that we can take care of cross-links by only solving intra-links and using the associated coreference relations. The only candidate arguments of cross-links are the arguments which are coreferent to intra-sentence mentions (antecedents).

The improvement due to Formula (*T*) depends on the accuracy of the intra-link role(*i*, *j*, *r*) and coreference relation corefer(*j*, *k*) atoms. Clearly, this accuracy depends partially on the effectiveness of Formula (*SiD*) above. It should also be clear that the improvement due to Formula (*SiD*) is also affected by Formula (*T*) because *T* impacts the condition ∃*i.*role(*i*, *j*, *r*) in Formula (*SiD*)*.* Thus, the formulae representing *Salience in Discourse* and *Transitivity* interact with each other.

#### Feature copy

We make additional use of coreference information through *“Feature Copy”.* The main idea is to supplement the features of an anaphor by adding the features of its antecedent. According to the example of Figure 1, the formula:

corefer(11, 4) ∧ word(4, “IRF-2”) ⇒ role(13, 11, Theme)

describes a word feature “IRF-2” to the anaphor “The region” in *S2.* Here word(*i*, *w*) represents a feature that the child token of the token *i* on the dependency subtree is word *w.* To be exact, this formula allows us to employ additional features of the antecedent to solve the link role(13, 11, Theme). This formula is generalized as Formula (*FC*) in the last row of Table 4. In Formula (*FC*), *F* denotes the predicates which represent basic features such as word, POS, and NE tags of the tokens. Formula (*FC*) copies the features of cross-sentence arguments (antecedents) to intra-sentence arguments (anaphors). *Feature Copy* is not a novel idea but it helps improve performance. For the SVM pipeline model we add equivalent features.

### Coreference resolution

In our work, we introduce a simple coreference resolver based on a pairwise coreference model [10]. It employs a binary classifier which classifies all possible pairs of noun phrases into “corefer” or “do not corefer”. Popular external resources like WordNet often do not work well in the biomedical domain. Hence, our resolver identifies coreference relations using only basic features such as word form, POS, and NE tag. We use SVM-struct for learning and testing the binary classifiers. In this model, negative examples often overwhelm positive ones, and we therefore select a value over 10000 for the *C*-parameter. We achieve 59*.*1 pairwise F1 on GENIA Event Corpus evaluating 5-fold cross validation.

There is some previous work on coreference resolution for biomedical domains [11,12]. They constructed original coreference annotations for learning and testing. Their models use much richer features for machine learning classifiers and their systems achieve better results with around 70 F1. However, owing to the differences of the data used, it is difficult to directly compare their results with ours. Moreover, using the richer feature they propose, we would likely see improvements in our system as well. Finally, we confirm that there is enough room for improvement by also evaluating with gold coreference annotations.

Note that we optimize our resolver for event extraction because our event extractors require high precision results from coreference resolution. For the SVM model, coreference resolution errors directly hurt performance. For MLN model, noisy results from coreference resolution often disturb the coreference formulae when learning weights. We noticed that the weights of coreference formulae remain small when the coreference resolution results have less than 70 precision and our MLN event extractor rarely obtains cross-sentence event-argument relations as a result. Some features and string distance metrics may enable us to better balance precision and recall, but we attach greater importance to precision. As a result, our high precision resolver achieves over 90 for precision but lower than 50 for recall.

## Results

Let us summarise the data and tools we employ. The data for our experiments is the *GENIA Event Corpus* (*GEC*) [1]. For feature generation, we employ the following tools. POS and NE tagging are performed with the *GENIA Tagger* (http://www-tsujii.is.s.u-tokyo.ac.jp/GENIA/tagger/), for dependency path features we apply the *Charniak-Johnson reranking parser with a Self-Training parsing model* (http://www.cs.brown.edu/~dmcc/biomedical.html), This model is optimized for biomedical parsing and achieves 84*.*3pt F1 on GENIA corpus [13]. We convert the parsed results to dependency tree using the *pennconverter tool* (http://nlp.cs.lth.se/software/treebank_converter/). Learning and inference algorithms for joint model are provided by *Markov thebeast*[14], a Markov Logic engine tailored for NLP applications. Our pipeline model employs *SVM-struct* (http://www.cs.cornell.edu/People/tj/svm_light/svm_struct.html) both in learning and testing. As we mentioned in the previous section, for coreference resolution, we also employ SVM-struct for binary classification.

**Figure 3:**
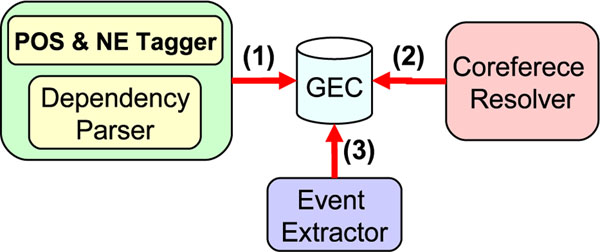
**Experimental setup**. An illustration of experimental setup. Data for learning and evaluation: GENIA Event Corpus (GEC). POS and NE Tagger: GENIA Tagger. Dependency Parser: Charniak-Johnson reranking parser with a Self-Training parsing model. Coreference Resolver: Pairwise model. Event Extractor: SVM-struct(SVM) and Markov TheBeast(MLN)

Figure 3 shows the structure of our experimental setup. Our experiments perform the following steps. (1) First we perform preprocessing (tagging and parsing). (2) Then we perform coreference resolution for all the documents and generate lists of token pairs that are coreferent to each other. (3) Finally, we train the event extractors: SVM pipeline (SVM) and MLN joint (MLN) involving coreference relations. We evaluate all systems using 5-fold cross validation on GEC.

In the following we will first show the results of our models for event extraction with/without coreference information. We will then present more detailed results concerning E-A relation extraction.

### Impact of coreference based approach

We begin by showing the SVM and MLN results for event extraction in Table 5. We present F1-values of event, eventType, and role (E-A relation) extraction. The three columns (event, eventType, and role) in Table 5 correspond to the *hidden* predicates in Table 2.

**Table 5:** Results of event extraction (F1)

System	Coreference	event	eventType	role
(a) SVM	NONE	77.0	67.8	52.3 ( 0.0)
(b) SVM	SYS	77.0	67.8	53.6 (**+1.3**)
(b′) SVM	GOLD	77.0	67.8	55.4 (+3.1)

(c) MLN	NONE	80.5	70.6	51.7 ( 0.0)
(g) MLN	SYS	80.8	70.8	53.8 (**+2.1**)
(g′) MLN	GOLD	81.2	70.8	56.7 (+5.0)

“Coreference” has the tree options: without coreference information (NONE), with coreference resolver (SYS), and with gold coreference annotations (GOLD)

Let us consider rows (a)-(b) and (c)-(g). They compare the SVM and MLN approaches with and without the use of coreference information. The column “Corefer” indicates how the coreference information is used: “NONE”–without coreference; “SYS”– with coreference resolver; “GOLD”– with gold coreference annotations.

We note that adding coreference information leads to 1.3 point F1 improvement for the SVM pipeline, and a 2.1 point improvement for MLN joint. Both improvements are statistically significant (*p* < 0*.*01, McNemar’s test 2-tailed).

With gold coreference information, systems (b′) and (g′) clearly achieve more significant improvements. Let us move on to the comparisons between SVM pipeline and MLN joint models. For event and eventType we compare row (b) with row (g) and observe that the MLN outperforms the SVM. This is to be contrasted with results for the BioNLP‘09 shared task, where the SVM model [3] outperformed the MLN [7]. This contrast may stem from the fact that GEC events are more difficult to extract due to a large number of event types and lack of gold protein annotations, and hence local models are more likely to make mistakes that global consistency constraints can rule out. For role extractions (E-A relation), SVM pipeline and MLN joint show comparable results, at least when not using coreference relations. However, when coreference information is taken into account, the MLN profits more. In fact, with gold coreference annotations, the MLN outperforms the SVM pipeline by a 1.3 point margin.

### Detailed results for event-argument relation extraction

Table 6 shows the three types of E-A relations we evaluate in detail.

**Table 6:** Three types of event-argument relations

Type	Description	Edge in Figure 1
Cross	E-A relations crossing sentence boundaries (cross-link)	Arrow (C)
W-ANT	Intra-sententence E-As (intra-link) with antecedents	Arrow (A)
Normal	Neither Cross nor W-ANT	Arrow (D)

They correspond to the arrows (A), (C), and (D) in Figure 1, respectively. We show the detailed results of E-A relation extraction in Table 7. All scores shown in the table are F1-values.

**Table 7:** Results of E-A relation extraction (F1)

System	Corefer	Cross	W-ANT	Normal
(a) SVM	NONE	0.0	56.0	53.6
(b) SVM	SYS	**27.9**	57.0	54.3
(b′) SVM	GOLD	**54.1**	57.3	55.4

(c) MLN	NONE	0.0	49.8 ( 0.0)	53.2
(d) MLN	*FC*	0.0	51.5 (+1.7)	53.7
(e) MLN	*FC*+*SiD*	0.0	54.6 (+4.8)	53.3
(f) MLN	*FC*+*T*	36.7	51.7 (+1.9)	53.7
(g) MLN	*FC*+*SiD*+*T*	**39.3**	56.5 (**+6.7**)	54.3
(g′) MLN	GOLD	**69.7**	66.7 (**+16.9**)	55.3

“Coreference” options include without coreference information (NONE), with coreference resolver (SYS), with gold coreference annotations (GOLD), with Feature Copy (FC), with Salience in Discourse (SiD), and with Transitivity (T)

#### SVM pipeline model

The first part of Table 7 shows the results of the SVM pipeline with/without coreference relations. Systems (a), (b) and (b′) correspond to the first three rows in Table 5, respectively. We note that the SVM pipeline manages to extract cross-links with an F1 score of 27.9 points with coreference information from the resolver. The third low in Table 7 shows the results of the system with gold coreference which is extended from System (b). With gold coreference, the SVM pipeline achieves 54.1 points for “Cross”. However, the improvement we get for “W-ANT” relations is small since the SVM pipeline model employs only *Feature Copy* and *Transitivity* concepts. In particular, it cannot directly exploit *Salience in Discourse* as a feature.

#### MLN joint model

How does coreference help our MLN approach? To answer this question, the second part of Table 7 shows the results of the following six systems. The row (c) corresponds to the fourth row of Table 7 and shows results for the system that does not exploit any coreference information. Systems (d)-(g) include Formula (*FC*). In the sixth (e) and the seventh (f) rows, we show the scores of MLN joint with Formula (*SiD*) and Formula (*T*), respectively. Our full joint model with both (*SiD*) and (*T*) formulae comes in the eighth row (g). System (g′) is an extended system from System (g) with gold coreference information.

By comparing Systems (d)(e)(f) with System (c), we note that *Feature Copy* (*FC*), *Salience in Discourse* (*SiD*), and *Transitivity* (*T*) formulae all successfully exploit coreference information. For “W-ANT”, Systems (d) and (e) outperform System (c), which establishes that both *Feature Copy* and *Salience in Discourse* are sensible additions to an MLN E-A extractor. On the other hand, for “Cross (cross-link)”, System (f) extracts cross-sentence E-A relations, which demonstrates that *Transitivity* is important, too. Next, for cross-link, our full system (g) achieved 39*.*3 points F1 score and outperformed System (c) with 6*.*7 points margin for “W-ANT”. The further improvements with gold coreference are shown by our full system (g′). It achieved 69*.*7 points for “Cross” and improved System (c) by 16*.*9 points margin for “W-ANT”.

#### SVM pipeline vs MLN joint

The final evaluation compares SVM pipeline and MLN joint models. Let us consider Tables 7 again. When comparing System (a) with System (c), we notice that the SVM pipeline (a) outperforms the MLN joint model in “W-ANT” without coreference information. However, when comparing Systems (b) and (g) (using coreference information by the resolver), MLN result is very competitive for “W-ANT” and 11*.*4 points better for “Cross”. Furthermore, with gold coreference, the MLN joint (System (g′) outperforms the SVM pipeline (System (b′)) both in “Cross” and “W-ANT” by a 15.6 points margin and a 9.4 points margin, respectively. This demonstrates that our MLN model will further improve extraction of cross-links and intra-links with antecedents if we have a better coreference resolver. Note that the MLN model has advantages over the SVM model especially when higher recall is required. We have 2, 124 links of “Cross” and 2, 748 of “W-ANT” for the evaluation of Table 7. MLN model-System (g′) finds 1, 236 correct “Cross” and 1, 778 correct “W-ANT” links. The SVM model-System (b′) finds only 833 correct links for “Cross” and 1, 149 for “W-ANT”. We believe that the reason for these results are two crucial differences between the SVM and MLN models:

• With Formula (*SiD*) in Table 4, MLN joint has more chances to extract “W-ANT” relations. It also effects the first term of Formula (*T*). By contrast, the SVM pipeline cannot easily model the notion of *salience in discourse* and the effect from coreference is weak.

• Formula (*T*) of MLN is defined as a soft constraint. Hence, other formulae may reject a suggested cross-link from Formula (*T*). The SVM pipeline deterministically identifies cross-links and is hence more prone to errors in the intra-sentence E-A extraction.

Finally, the potential for further improvement through a coreference-based approach is limited by the performance on intra-links extraction. Moreover, we also observe that the 20% of cross-links are cases of zero-anaphora. Here the utility of coreference information is naturally limited, and our Formula (*T*) cannot come into effect due to missing corefer(*j*, *k*) atoms.

## Conclusions

In this paper we presented a novel approach to event extraction with the help of coreference relations. Our approach incorporates coreference relations through the concepts of *salience in discourse* and *transitivity*. The coreferent arguments we focused on are generally valuable for document understanding in terms of discourse structure and they should be extracted at all cost. We proposed two models: SVM pipeline and MLN joint. Both improved the attachments of intra-sentence and cross-sentence related to coreference relations. Furthermore, we confirmed that improvements of coreference resolution lead to the higher performance of event-argument relation extraction. However, potential for further improvement through a coreference-based approach is limited by the performance of intra-sentence links and zero-anaphora cases. To overcome these problems, we plan to investigate a collective approach that works on the full document. Specifically, we are constructing a joint model of coreference resolution and event extraction considering all tokens in a document based on the idea of Narrative Schemas [15]. If we take into account all tokens in a document at the same time, we can consider various relations between events (event chains) through anaphoric chains. But to implement such a joint model in Markov Logic, we will have to cope with the time and space complexities that arise in such a setting. We are now investigating reasonable approximations for learning and inference of such joint models.

## Competing interests

The authors declare that they have no competing interests.

## Authors' contributions

KY and TH participated in the design of the study and ran the algorithms for analysis. SR implemented the Markov Logic Engine and helped with the design of the basic approach. KY, SR and TH primarily wrote the manuscript. MA and YM helped with the analysis and wrote parts of the manuscript. All authors read and approved the final manuscript.
